# Long non-coding RNA HOXA11-AS regulates ischemic neuronal death by targeting miR-337-3p/YBX1 signaling pathway: protective effect of dexmedetomidine

**DOI:** 10.18632/aging.204648

**Published:** 2023-04-10

**Authors:** Fei Yan, Pinxiao Wang, Xiaojian Yang, Fuli Wang

**Affiliations:** 1 School of Pharmacy, Health Science Center, Xi’an Jiaotong University, Xi’an, Shaanxi 710115, China; 2 Department of Urology, Xi’an Medical University, Xi’an, Shaanxi 710068, China; 3 Department of Urology, Xijing Hospital, Fourth Military Medical University, Xi’an, Shaanxi 710000, China

**Keywords:** ischemia stroke, apoptosis, dexmedetomidine, miR-337-3p, long non-coding RNA

## Abstract

Cerebral ischemia/reperfusion (I/R) is a common neurological disease. Homeobox A11 antisense RNA (HOXA11-AS), a long non-coding RNA (lncRNA), has been demonstrated as an important regulator in diverse human cancers. However, its function and regulatory mechanism in ischemic stroke remains largely unknown. Dexmedetomidine (Dex) have received wide attraction because of its neuroprotective effects. This study aimed to explore the possible link between Dex and HOXA11-AS in protecting neuronal cells from by ischemia/reperfusion-induced apoptosis. We used oxygen–glucose deprivation and reoxygenation (OGD/R) in mouse neuroblastoma Neuro-2a cells and middle cerebral artery occlusion (MACO) mouse model to test the link. We found that Dex significantly alleviated OGD/R-induced DNA fragmentation, cell viability and apoptosis, and rescued the decreased HOXA11-AS expression after ischemic damage in Neuro-2a cells. Gain-/loss-of-function studies revealed that HOXA11-AS promoted proliferation, inhibited apoptosis in Neuro-2a cells exposed to OGD/R. Knockdown of HOXA11-AS decreased the protective effect of Dex on OGD/R cells. HOXA11-AS was found to transcriptionally regulate microRNA-337-3p (miR-337-3p) expression as evidenced by luciferase reporter assay, while miR-337-3p expression was upregulated following ischemia *in vitro* and *in vivo*. Besides, knockdown of miR-337-3p protected OGD/R-induced apoptotic death of Neuro-2a cells. Furthermore, HOXA11-AS functioned as a competing endogenous RNA (ceRNA) and competed with Y box protein 1 (Ybx1) mRNA for directly binding to miR-337-3p, which protected ischemic neuronal death. Dex treatment protected against ischemic damage and improved overall neurological functions *in vivo*. Our data suggest a novel mechanism of Dex neuroprotection for ischemic stroke through regulating lncRNA HOXA11-AS by targeting the miR-337-3p/Ybx1 signaling pathway, which might help develop new strategies for the therapeutic interventions in cerebral ischemic stroke.

## INTRODUCTION

Cerebral ischemia/reperfusion (I/R) is a common neurological disease caused by brain injury with high mortality and disability, and there is still no effective treatment [1]. Age is an important risk factor for stroke [2]. Therefore, it is necessary to explore innovative treatments, particularly for the elderly people with a high incidence of stroke. Cerebral I/R are characterized by initially limiting blood supply to the brain, then restoring blood flow and simultaneously reoxygenating [3, 4]. It involves brain tissue death or cerebral infarction caused by insufficient blood and oxygen supply due to cerebral vascular occlusion. Cerebral I/R-induced cerebrovascular dysfunction is considered to be an important cause of nervous system damage in diseases such as ischemic stroke. Finding effective measures to prevent neuronal injury is of great significance in preventing ischemic stroke. Although timely ischemia-reperfusion can prevent severe neurological deficits and apoptosis/necrosis, it may further aggravate neuronal apoptosis/necrosis and neurological deficits [4]. Therefore, strategies for minimizing the I/R-induced injuries are urgently needed.

Long noncoding RNAs (lncRNAs), usually with little or no protein-coding capacity, are longer than 200 bp and still have inherent functions as RNA [5]. Emerging evidence has verified that lncRNAs are involved in a variety of biological processes like cell differentiation, proliferation, apoptosis and invasion [6–8]. LncRNAs are reported to positively or negatively regulate target gene expression in many human diseases at different levels, including chromatin modification, epigenetic, transcriptional and post-transcriptional modification [9–11].

Homeobox A11 antisense RNA (HOXA11-AS), located in the HOXA gene cluster on chromosome 7p15.2, is a highly conserved lncRNA and originally found in mouse embryo cDNA libraries [12, 13]. HOXA11-AS, a novel regulator in human cancer proliferation and metastasis, is involved in many types of cancer progression [14–16]. For example, HOXA11-AS has been reported to promote cell migration and invasion in gastric cancer by regulating β-catenin and KLF2 [17]. In addition, HOXA11-AS can also promote retinoblastoma progression via sponging miR-506-3p [18]. The connection between HOXA11-AS and apoptosis has been well documented in multiple cell types, including ovarian cancer cells [19], non-small cell lung cancer [20], melanoma cells [21], and human glioma cells [22]. However, little is known about the biological function and possible molecular mechanisms of HOXA11-AS in ischemic stroke-associated apoptotic neuronal cell death. Therefore, one of the objectives of this study is to study the biological function of HOXA11-AS in ischemic stroke and its underlying molecular mechanism. MicroRNA-337-3p (miR-337-3p) was predicted as a potential target of HOXA11-AS by bioinformatics analysis. A recent study demonstrated that a chemically modified antimir-337-3p penetrated the blood–brain barrier (BBB), functionally incorporated into neurons, down-regulated complementary miRNA, and mediated neuroprotection in the setting of cerebral ischemia [23]. Y box binding protein 1 (Ybx1) is a DNA/RNA--binding protein is known to be involved in various types of cancers [24]. Ybx1 was predicted as a target gene of miR-337-3p according to our bioinformatic analysis. Therefore, we focused on these molecules and explore their connections with stroke-induced apoptotic neuronal cell death.

Dexmedetomidine (Dex), a selective agonism of alpha2 (α2)-adrenergic receptors, is applied for sedation and analgesia in clinical patients. Recently, Dex has received widespread attention for its neuroprotective effects. It turned out that Dex prevented mice brain damage caused by subarachnoid hemorrhage through activating mitogen-activated protein kinase (MAPK) [25, 26]. In rats with intracerebral hemorrhage, Dex reduced short-term and spatial learning and memory deficits by enhancing brain-derived neurotrophic factor expression and inhibiting apoptosis [27]. Dex has been reported to prevent I/R-induced intestinal, myocardial, renal, pulmonary, brain and liver damage [28]. Another study showed the potential benefit of Dex for cerebral infarction caused by obstruction of middle cerebral artery in rats [29]. However, the effect of Dex on I/R-induced neuronal damage is still unclear. In this study, using oxygen–glucose deprivation and reoxygenation (OGD/R) in mouse neuroblastoma Neuro-2a cells and the middle cerebral artery occlusion (MACO) mouse model, we tested the possible link between Dex and lncRNA-HOXA11-AS in protecting neuronal cells from I/R-induced apoptosis, and explored the underlying mechanisms.

## MATERIALS AND METHODS

### Cell I/R stress model establishment and drug treatment

Neuro-2a cells were obtained from Cell Bank Chinese Academy Science (CAS, Shanghai, China). Cells were cultured in Dulbecco’s medium (DMEM, Gibco, USA) supplemented with 10% heat-inactivated fetal bovine serum (FBS), L-glutamine, 100 IU/ml penicillin, and 100 μg/ml streptomycin in a 37°C incubator with a humidified atmosphere of 5% CO_2_. Dex (TOYOBO, Shanghai, China), dissolved in DMEM, was added into cell cultures at the doses of 5, 10, and 20 μM for 24 hours, and the dose of 10 μM was also applied for 3, 6, 12, and 24 hours to test its toxicity. The cells received equal volume of DMEM only were considered as the control group. Cell I/R stress model was built by oxygen and glucose deprivation as previous literature [30]. Glucose-free DMEM and phosphate-buffered saline (PBS) were bubbled with 100 % N_2_ for 30 min to exclude oxygen. After washing twice with buffer, Neuro-2a cells were seeded and cultured in glucose-free DMEM for 3 hours, and then treated with perfusion for 24 hours.

MTT assay was used to detect cell viability. Briefly, neuro-2a cells were seeded in 96-well tissue culture plates at the density of 1 × 10^4^ cells per well for 24 hours. Cells were treated with the drug at designed time and dose. Then, 0.5 mg/ml 3-(4,5-dimethylthiazol-2-yl)-2,5-diphenyltetrazolium bromide was added into the culture medium for a further 3 hours’ incubation to detect cell viability. The blue formazan products were dissolved in dimethyl sulfoxide (DMSO) and the optical density values at 490 nm (OD_490_) were measured with a microplate reader.

### Quantification of DNA fragmentation

DNA fragmentation was quantified using a cellular DNA fragmentation enzyme-linked immunosorbent assay (ELISA) kit (Millipore-Sigma, Billerica, MA, USA) according to the manufacturer’s protocol. Briefly, Neuro-2a cells labeled with bromodeoxyuridine (5-bromo-2’-deoxyuridine) (BrdU) were subcultured in 24-well tissue culture plates at a density of 2 × 10^5^ cells per well overnight. After harvested and suspended in culture medium, one hundred microliters were added to each well of 96-well tissue culture plates. After drug treatment, cells were seeded at 37°C in a humidified atmosphere of 5% CO_2_. Amounts of BrdU-labeled DNA in the cytoplasm were detected with anti-BrdU antibody conjugated with peroxidase. DNA fragmentation was quantified as optical density values at an optimal wavelength of 405 nm using a microplate photometer (GloMax^®^-96 Microplate Luminometer, Promega).

### Analysis of apoptotic cells

Cell apoptosis was quantified using propidium iodide (PI) and Annexin V staining. After Dex and I/R treatments, Neuro-2a cells were subjected to flow cytometric analysis to detect apoptosis using the Annexin V-fluorescein isothiocyanate (FITC)/PI Apoptosis Kit (BD Biosciences, San Jose, CA, USA) according to the manufacturer’s protocol. Briefly, after washing with PBS and the binding buffer for one time each, cells were stained with Annexin V-FITC/PI for 20 min at room temperature in dark. After washing with the binding buffer once, the labeled cells were detected immediately by a flow cytometer (Beckman Coulter, Franklin Lakes, NJ, USA). The percentage of apoptotic cells was defined as the % of Annexin V-positive cells (including both PI-negative and PI-positive populations) among the whole cells.

### RNA extraction and qRT-PCR analysis

Total RNA and miRNAs were extracted from brain infarction tissues, or brain tissues after HOXA11-AS or Dex injection, or differently conditioned neuro-2a cells with or without transfection of si-HOXA11-AS, PcDNA3.1-HOXA11-AS (Gene-Pharma, Shanghai, China) using TRIzol reagent (Invitrogen, Carlsbad, CA, USA) and miRNeasy Mini Kit (Qiagen, Hilden, Germany), respectively. For miRNA analysis, cDNA was obtained using the TaqMan MicroRNA Reverse Transcription Kit (Applied Biosystems, Foster City, CA, USA) and qRT-PCR was performed using TaqMan miRNA assay kit (Applied Biosystems). U6 small nuclear RNA was used as an endogenous control for normalization. For mRNA analysis, cDNA was synthesized by using M-MLV reverse transcriptase (Invitrogen) and reverse transcription primers Oligo (dT). qRT-PCR was then performed with SYBR Green Real-Time PCR Master Mixes (Thermo-Fisher, Waltham, MA, USA) on a PCR machine (Applied Biosystems).

### Luciferase reporter assay

The targeting relationship between HOXA11-AS and miRNAs, miR-337-3p targeted candidate genes and the binding sites of miR-337-3p were predicted using the online tools miRDB (https://mirdb.org/), TargetScan (http://www.targetscan.org/), and microT (https://dianalab.e-ce.uth.gr/html/dianauniverse/index.php?r=microT_CDS). Before luciferase reporter assay, pmirGLO- HOXA11-AS-WT, and pmirGLO-HOXA11-AS-MUT were constructed by GenePharma (Gene-Pharma, Shanghai, China). miR-337-3p mimics, mimic control, miR-337-3p inhibitor and a negative control of scramble RNA were also from GenePharma (Gene-Pharma, Shanghai, China). PcDNA3.1-HOXA11-AS, Si-HOXA11-AS and si-s-HOXA11-AS plasmids were designed and cloned into a pcDNA3.0 vector. Luciferase activity was measured when cells were harvested 48 hours post-transfection with the Dual Luciferase Reporter Assay System (Promega, Madison, WI, USA) according to the manufacturer’s instructions.

### Western blot

Cultures cells or brain samples were lysed and quantified. Protein samples (20 μg) were separated by 10% sodium dodecyl-sulfate polyacrylamide gel electrophoresis (SDS-PAGE) gel and then transferred to polyvinylidene difluoride (PVDF) membranes. After blocking for 2 hours with 5% bovine serum albumin (BSA) in Tris Buffered Saline with Tween 20 (TBST) and 0.1% Tween 20, primary antibodies against Ybx1 and β-actin (1:1000, Miliporesigma, Billerica, MA, USA) were subsequently added and incubated with the membrane for 16 hours at 4°C. After washing 3 times with TBST, the secondary antibody (1:5000 dilution; Miliporesigma, Billerica, MA, USA) was added to the membrane and the membrane was incubated for 1 hour at room temperature. Protein bands were detected by enhanced chemiluminescence and analyzed by Image-J software.

### Animals and middle cerebral artery occlusion (MACO) model

C57BL/6 J male mice (3 months old, 25–28 g) were obtained from Vital River Laboratory Animal Technology Co., Ltd. (Beijing, China) and kept in a humidity and temperature-regulated room with the cycle of 12-h light and 12-h dark. The anesthetized animal was placed under an operating microscope to make a midline incision. Right common carotid artery (CCA), external carotid artery (ECA), and internal carotid artery (ICA) were exposed. A 6/0 surgical nylon monofilament with a rounded tip was induced into the lumen of ICA gently from the right ECA until the rounded tip blocked the origin of the middle cerebral artery (MCA). After 60 min of MCAO, the surgical nylon monofilament was gently retracted to allow reperfusion for 24 hours. The sham-operated animals were treated with similar operations except without surgical nylon monofilament insertion. Mice were kept on a heating panel which maintained the temperature at 37°C during operation. TTC (2,3,5-triphenyltetrazolium chloride) staining was performed for visualization of the brain infarction, as previously reported [31]. The infarct volume of I/R injury and the volume of the whole brain were determined. The infarct area (%) was calculated according to the ratio of infarct volume to the whole volume of brain. The use and care of animals were performed in accordance with the guidelines for the Care and Use of Laboratory Animals of Xijing Hospital, Fourth Military Medical University and approved by the Institutional Animal Care and Use Committee.

### Stereotaxic injection of HOXA11-AS mimics in mice and drug administration

Mice in the HOXA11-AS group were anesthetized and fixed to a stereotaxic apparatus. HOXA11-AS mimics was diluted with the same volume of *in vivo* transfection reagent (Entranster™-*in vivo*; Engreen, Beijing, China) and administered into the lateral ventricle of mice. The stereotaxic coordinates were as follows: 0.6 mm posterior to the bregma, 1.0 mm lateral to the midline, and 2.5 mm under the dura. After one day injection, MCAO operation was conducted. Mice in the Dex group were administrated with Dex diluted with physiological saline.

### Modified neurological severity score test

Neurological function was assessed using the modified neurological severity score (mNSS) method [32]. The tests were conducted by observers who were blinded to the experiments. Neurological function including motor, sensory, balance and reflex tests was graded on a scale of 0 to 18 (normal score, 0; maximal deficit score, 18). 1 score point was awarded for the inability to perform the test or for the lack of a tested reflex; thus, the higher score, the more severe was the injury.

### Corner test

The corner test was performed to detect unilateral abnormalities of sensory and motor functions in the MACO mouse model, largely following the previous report [33]. The mouse was places in two pieces of 30 cm × 20 cm × 1 cm wooden board. The two wooden boards were placed at 30° angle, and there was a small gap between the two boards. The mouse was encouraged to enter this corner and was placed facing the corner. The mouse moved forward or upward and eventually turned left or toward turning right back. Cerebral ischemia mice preferentially turned back to the non-injured side, ipsilateral (right) side each time. Numbers of corners visited were recorded during 300 seconds.

### Statistical analysis

Data were expressed as mean ± standard deviation (SD). SPSS software (version 20; IBM, Armonk, NY, USA) and one-way Analysis of Variance (ANOVA) were used for statistical analysis. A *P* value smaller than 0.05 was considered statistically significant.

### Availability of data and materials

The datasets used and/or analyzed during the present study are available from the corresponding author upon reasonable request.

## RESULTS

### Dex protected Neuro-2a cells from I/R-induced apoptotic death

**Figure 1:**
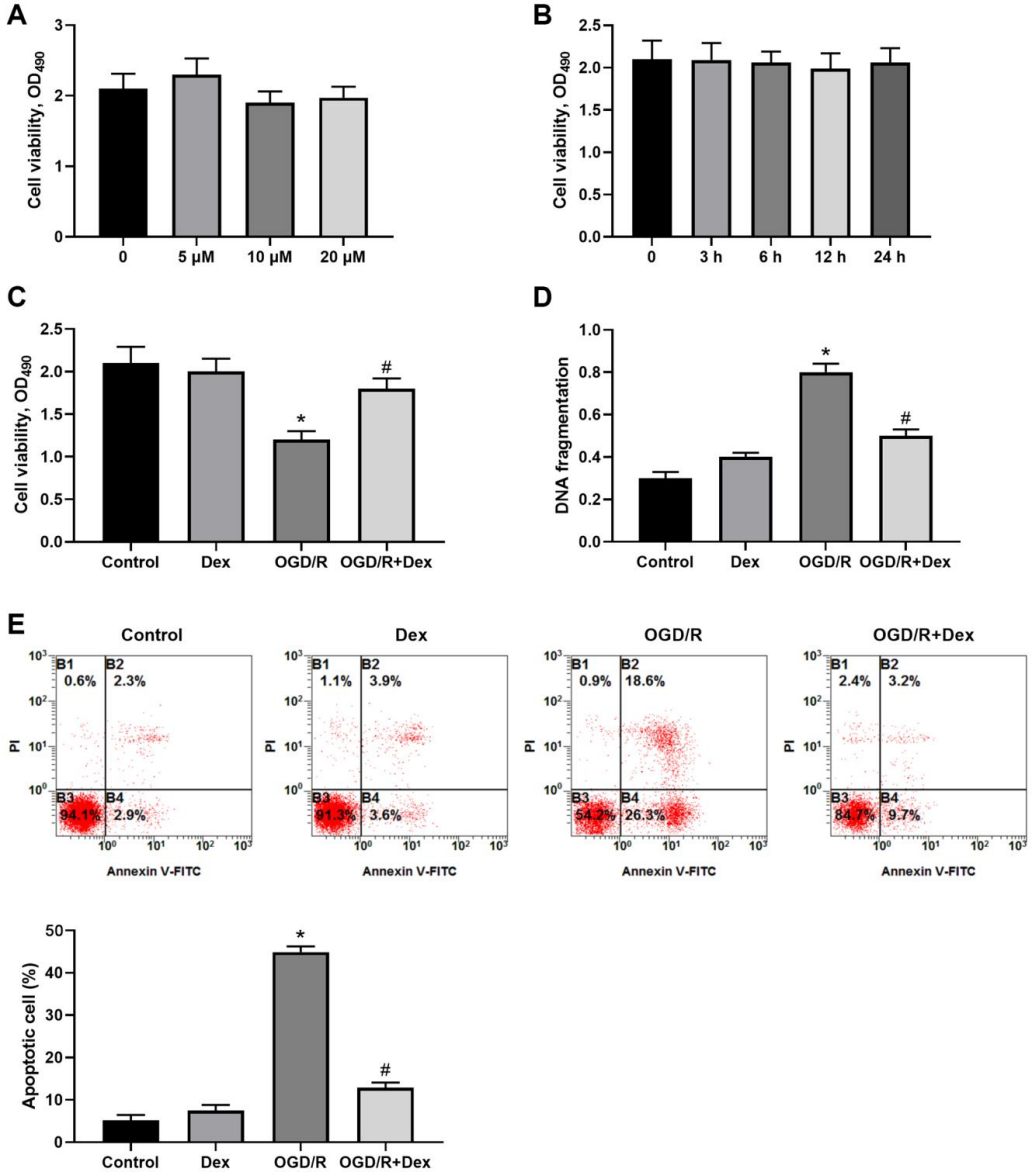
**Protective effects of dexmedetomidine (Dex) on ischemia/reperfusion (I/R)-induced neuronal death.** (**A**) Neuro-2a cells were exposed to 5, 10, 20 μM Dex for 24 h, and cell viability was measured. (**B**) Neuro-2a cells were treated with 10 μM Dex for 3, 6, 12, and 24 h, and cell viability was measured. (**C**–**E**) The effects of Dex on I/R-induced insults. Neuro-2a cells were treated with 10 μM Dex, received ischemia for 3 h then reperfusion for 24 h (I/R). Cell viability was analyzed (**C**), and DNA fragmentation was quantified with an ELISA method (**D**). The apoptotic cells were quantified by Annexin V/PI staining and statistically analyzed (**E**). *n* = 3 for each group; ^*^P < 0.05, compared with the Control group; ^#^*P* < 0.05, compared with the OGD/R group.

Neuro-2a cells were treated with Dex at the doses of 5 μM, 10 μM, and 20 μM for 24 h, or at the dose of 10 μM for 3, 6, 12, 24 h to evaluate drug toxicity. The results showed that the cells viability had no much changes, indicating that Dex had no cytotoxicity in these doses (Figure 1A) and time range (Figure 1B). Neuro-2a cells were exposure to OGD for 3 h and then subjected to reperfusion for 24 h (OGD/R) significantly decreased cell viability by about 40% (Figure 1C). To determine the effects of Dex on I/R-induced insults to neuronal cells, Neuro-2a cells were treated with 10 μM Dex before ischemia for 3 h then received reperfusion for 24 h (I/R). Dex rescued cell viability, which increased from 60.5% of control in the OGD/R alone group to 81.8% of control in the OGD/R+Dex group (Figure 1C). In addition, Neuro-2a cells exposed to I/R led to a 2.6-fold stimulations of DNA fragmentation, and additional administration of Dex deceased I/R-induced DNA fragmentation by 44.5% (Figure 1D). Moreover, exposure to I/R caused cell apoptosis and additional Dex treatment lowed the percentages of apoptotic cells from 44.9% to 12.9% (Figure 1E). Thus, Dex could protect Neuro-2a cells from I/R-induced apoptotic cell death.

### Dex treatment protected OGD/R exposed Neuro-2a cells through up-regulation of HOXA11-AS expression and increased cell survival

**Figure 2:**
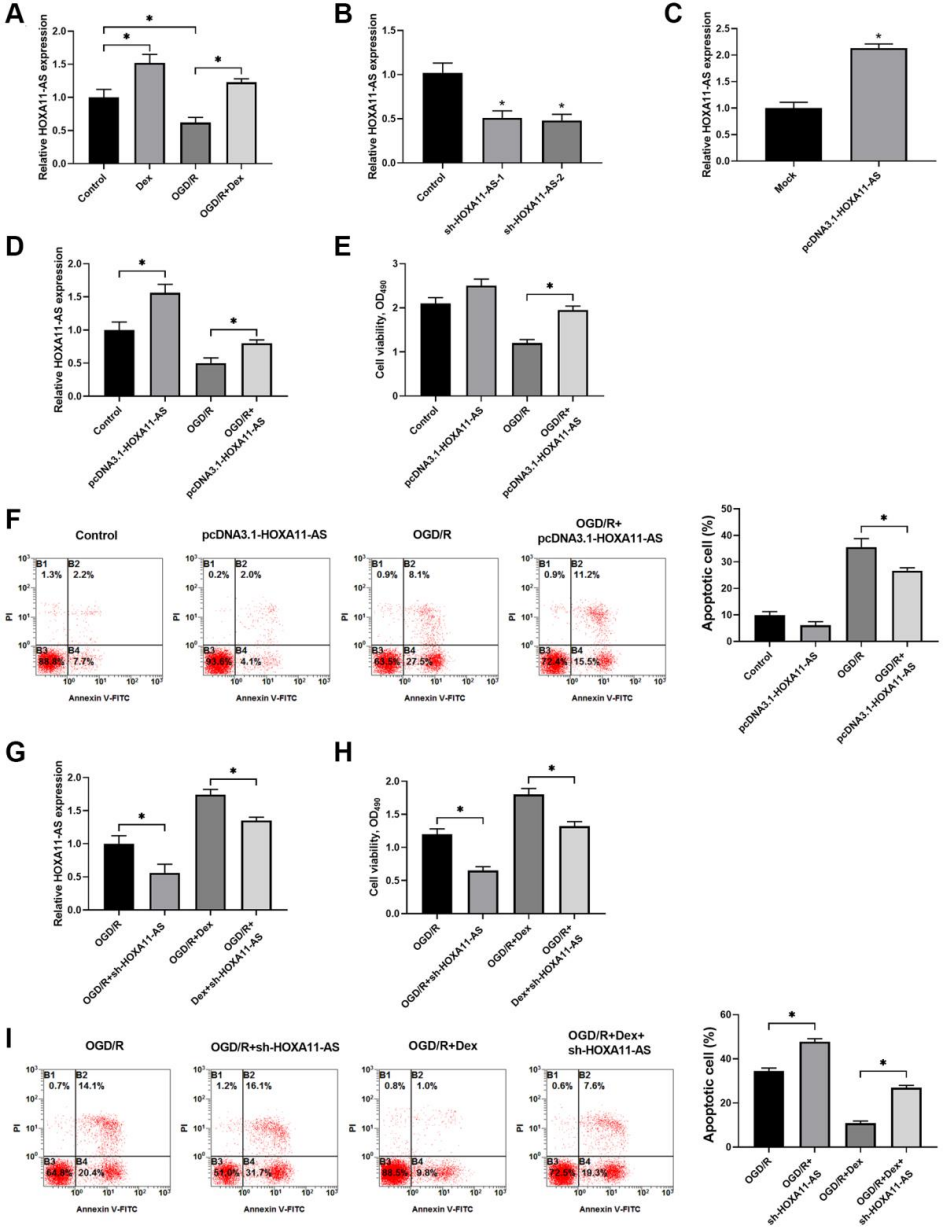
**Dex treatment protected OGD/R-exposed Neuro-2a cells through up-regulation of HOXA11-AS expression and increased cell survival.** (**A**) Neuro-2a cells with or without OGD/R were pre-treated with 10 μM Dex. The expression of HOXA11-AS was determined by quantitative real-time PCR. (**B**, **C**) Knockdown (**B**) and over-expression (**C**) of HOXA11-AS by transfection of sh-HOXA11-AS and pcDNA3.1-HOXA11-AS, respectively, were verified by quantitative real-time PCR. (**D**–**F**) Neuro-2a cells were transfected with control empty vector or pcDNA3.1-HOXA11-AS vector, and were left untreated or received OGD/R. Relative expression of HOXA11-AS (**D**), cell proliferation (**E**) and apoptosis of cells (**F**) in the indicated groups were quantitated. (**G**–**I**) Neuro-2a cells transfected with control sh-RNA or sh-HOXA11-AS were left untreated or treated with 10 μM Dex. Cells were then subjected to OGD/R. Relative expression of HOXA11-AS (**G**), cell proliferation (**H**) and apoptosis of cells (**I**) in the indicated groups were quantitated. *n* = 3 for each group; ^*^P < 0.05, compared between the indicated groups.

The expression of HOXA11-AS was determined by quantitative real-time PCR in the cells after OGD/R. Dex treatment alone significantly up-regulated HOXA11-AS expression and OGD/R decreased OXA11-AS expression in Neuro-2a cells. Administration of Dex also inhibited the decrease of OXA11-AS expression induced by OGD/R (Figure 2A). To investigate the functional mechanisms of HOXA11-AS in OGD/R, we used sh-HOXA11-AS and pcDNA3.1-HOXA11-AS to down-regulate its expression (Figure 2B) and over-express HOXA11-AS (Figure 2C), respectively. Transfection of pcDNA3.1-HOXA11-AS upregulated HOXA11-AS expression in cells without or with exposure to OGD/R (Figure 2D). MTT results revealed that HOXA11-AS overexpression rescued cell viability of OGD/R-exposed Neuro-2a cells, compared with the OGD/R alone groups (Figure 2E). Transfection of pcDNA3.1-HOXA11-AS also decreased the percentage of apoptotic cells induced by OGD/R (Figure 2F). To further explore the effects of Dex and HOXA11-AS knockdown on cells viability and apoptosis, we administrated Dex and performed sh-HOXA11-AS transfection to OGD/R treated cells. Compared with the OGD/R alone group and the OGD/R+Dex group, the groups with additional down-regulated HOXA11-AS expression (Figure 2G) showed decreased cell viability (Figure 2H) and increased cell apoptosis in OGD/R treated cells (Figure 2I), indicating that Dex exerted protective effects on OGD/R exposed Neuro-2a cells at least partially through up-regulation of HOXA11-AS expression.

### HOXA11-AS competed binding to miR-337-3p to silence miR-337-3p and inhibit apoptosis

**Figure 3:**
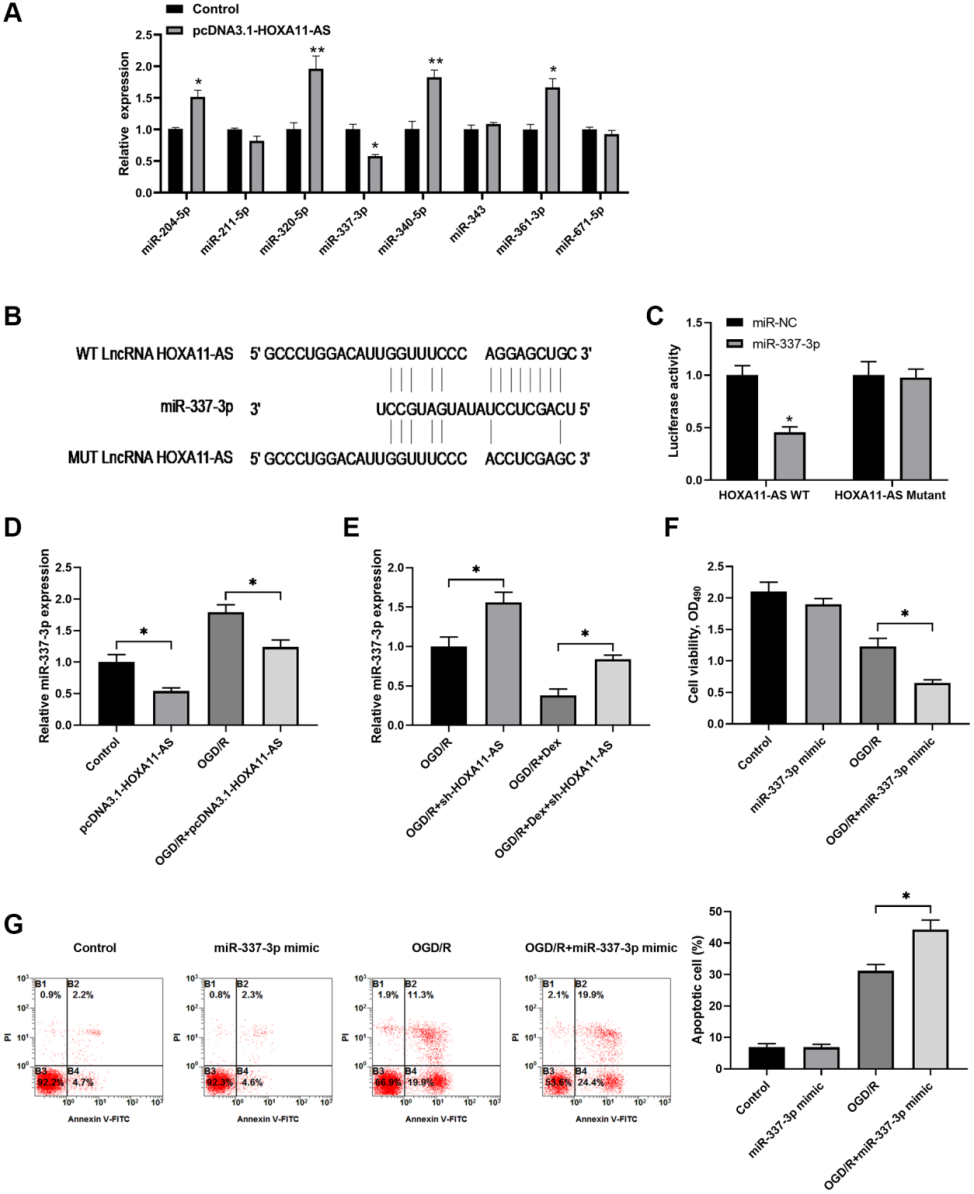
**HOXA11-AS competed binding to miR-337-3p to silence miR-337-3p and inhibit apoptotic cell.** (**A**) Neuro-2a cells were transfected with control empty vector or pcDNA3.1-HOXA11-AS vector, and the expression of candidate miRNAs was quantitated by quantitative real-time PCR. (**B**) Schematic diagram shows the HOXA11-AS transcript binding sites on miR-337-3p. The wild type (WT) and mutated (MUT) binding sites of HOXA11-AS are shown. (**C**) The luciferase activity of the reporter vectors was detected in Neuro-2a cells at 48 hours after co-transfection of the plasmid expressing wild type or mutant HOXA11-AS together with the NC mimic or the miR-337-3p mimic. (**D**, **E**) Neuro-2a cells were transfected with control empty vector or pcDNA3.1-HOXA11-AS vector, and were left untreated or received OGD/R (**D**). Neuro-2a cells transfected with control sh-RNA or sh-HOXA11-AS were left untreated or treated with 10 μM Dex. Cells were then subjected to OGD/R (**E**). The relative expression of miR-337-3p was quantitated. (**F**, **G**) Neuro-2a cells transfected with control miRNA mimics or miR-337-3p mimics were left untreated or received OGD/R. Cell proliferation (**F**) and apoptosis (**G**) in the indicated groups were measured. ^*^P < 0.05, ^**^P < 0.01, compared with the Control group or between the indicated groups.

Previous studies have demonstrated that lncRNAs could be involved in different physiology and pathology processes by functioning as competing endogenous RNAs (ceRNAs) of specific miRNAs [5, 6, 11]. Thus, we attempted to investigate the potential interaction between HOXA11-AS and miRNA in OGD/R. We used online tools to predict miRNAs targeted by HOXA11-AS. We screened 8 candidate miRNAs in the Neuro-2a cells transfected with pcDNA3.1-HOXA11-AS, and found that miR-337-3p expression was significantly inhibited by HOXA11-AS overexpression (Figure 3A). HOXA11-AS transcript binding sites on miR-337-3p were analyzed by bioinformatics database starBase v2.0 (Figure 3B), and a dual-luciferase reporter assay was performed to confirm the potential interaction between HOXA11-AS and miR-337-3p. The results showed that miR-337-3p mimics significantly decreased the luciferase activity of Neuro-2a cells transfected with the luciferase reporter vector HOXA11-AS-WT, while no obvious change was observed in luciferase activity of cells transfected with HOXA11-AS-MUT vector (Figure 3C). Furthermore, relative expression of miR-337-3p in Neuro-2a cells transfected with pcDNA3.1-HOXA11-AS or sh-HOXA11-AS was detected by qRT-PCR. HOXA11-AS overexpression markedly decreased the expression of miR-337-3p in cells with or without OGD/R (Figure 3D). HOXA11-AS knockdown increased miR-337-3p expression in OGD/R treated cells, irrespective of the existence of additional Dex treatment (Figure 3E). As shown in Figure 3F, 3G, additional miR-337-3p overexpression through miR-337-3p mimics transfection significantly decreased cell viability and increased cell apoptosis in OGD/R exposed cells, compared to the cells received OGD/R alone. Taken together, HOXA11-AS competed binding to miR-337-3p to silence miR-337-3p and inhibit apoptosis, which was one of the mechanisms underlying Dex’s neuroprotection effects.

### Dex protected neuronal cells from I/R-induced apoptosis through regulating the downstream effector YBX1 of the HOXA11-AS/miR-337-3p axis

**Figure 4:**
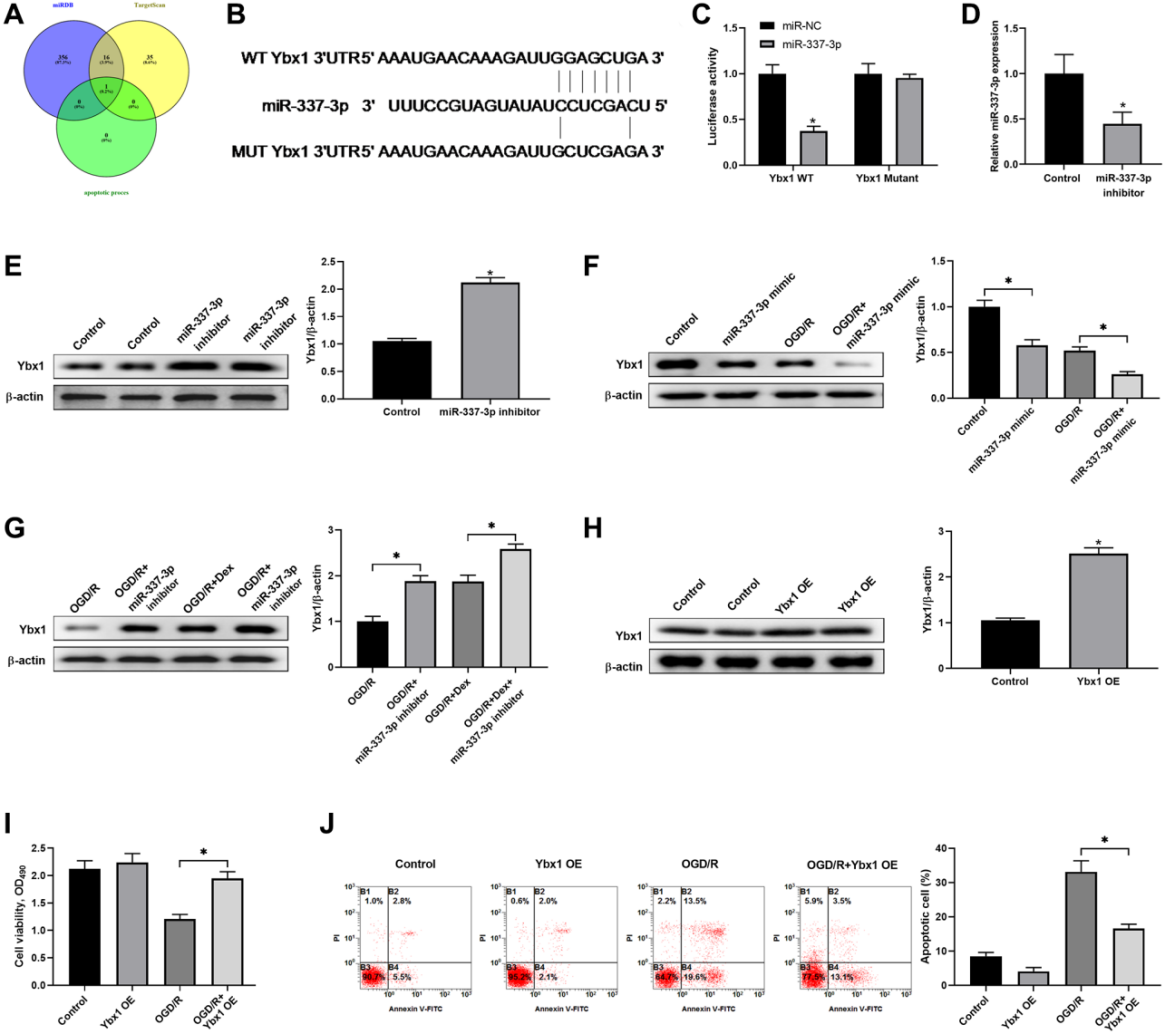
**Dex protected neuronal cells from apoptosis induced by ischemia/reperfusion through regulating YBX1 expression.** (**A**) Venn diagram shows the numbers of overlapping genes among the predicted genes of miR-337-3p using the online algorithms including miRDB, TargetScan and microT. (**B**) Schematic diagram shows the matching base pairs between miR-337-3p and the wild type (WT) or mutated (MUT) 3′-UTR of Ybx1 mRNA. (**C**) The luciferase activity of the reporter vectors was detected in Neuro-2a cells at 48 hours after co-transfection of the plasmid expressing wild type or mutant 3′-UTR of Ybx1 mRNA together with the NC mimic or the miR-337-3p mimic. (**D**, **E**) Neuro-2a cells were transfected with control miRNA inhibitor or miR-337-3p inhibitor, and the expression of miR-337-3p (**D**) and protein level of Ybx1 (**E**) were quantitated at 48 hours after transfection. (**F**) Neuro-2a cells were transfected with control miRNA mimics or miR-337-3p mimics, and left untreated or received OGD/R. The protein level of Ybx1 was quantitated. (**G**) Effects of miR-337-3p inhibitor transfection and Dex treatment, alone or in combination, in OGD/R-treated Neuro-2a cells on the expression of Ybx1 protein. (**H**) Confirmation of Ybx1 overexpression in Neuro-2a cells at 48 hours after transfection of Ybx1-overexpression (OE) plasmid. (**I**, **J**) Control cells or Ybx1-OE cells were left untreated or received OGD/R. Cell proliferation (**I**) and apoptosis (**J**) in the indicated groups were measured. ^*^P < 0.05, compared with the Control group or between the indicated groups.

As shown in Figure 4A, Venn diagram shows the numbers of overlapping genes among the predicted genes using the online algorithms including miRDB, TargetScan and microT. We found that miR-337-3p might bind to 3′-UTR of Ybx1 mRNA by bioinformatics analysis (Figure 4B). Luciferase reporter assay was conducted to confirm Ybx1 was a direct target of miR-337-3p. The plasmids of Ybx1-WT or Ybx1-MUT was co-transfected with miR-337-3p mimics into Neuro-2a cells. The results showed that miR-337-3p mimics significantly decreased the luciferase activity of cells transfected with the Ybx1-WT vector, but not that of Ybx1-MUT (Figure 4C). Compared to the negative control group, the group with knockdown of miR-337-3p by miR-337-3p inhibitor had significantly decreased miR-337-3p expression (Figure 4D) and significantly increased Ybx1 protein expression (Figure 4E). Overexpression of miR-337-3p by miR-337-3p mimics decreased Ybx1 protein expression in both the cells with or without OGD/R, and the different was that OGD/R made the decrease much severely (Figure 4F). Under OGD/R, Dex and miR-337-3p inhibitor, administrated separately or together, increased Ybx1 protein expression in cells; however, the treatment with Dex and miR-337-3p inhibitor together resulted in a much more increase of Ybx1 protein expression (Figure 4G). In addition, Ybx1 overexpression (Figure 4H) enhanced cell viability (Figure 4I) and reduced cell apoptosis (Figure 4J) induced by OGD/R. These results showed that HOXA11-AS competed with Ybx1 mRNA for binding to miR-337-3p, and Dex protected neuronal cells from ischemia/reperfusion-induced apoptosis through regulating the downstream effector Ybx1 protein.

### Dex/HOXA11-AS protected against ischemic damage and improved neurological deficits *in vivo*

**Figure 5:**
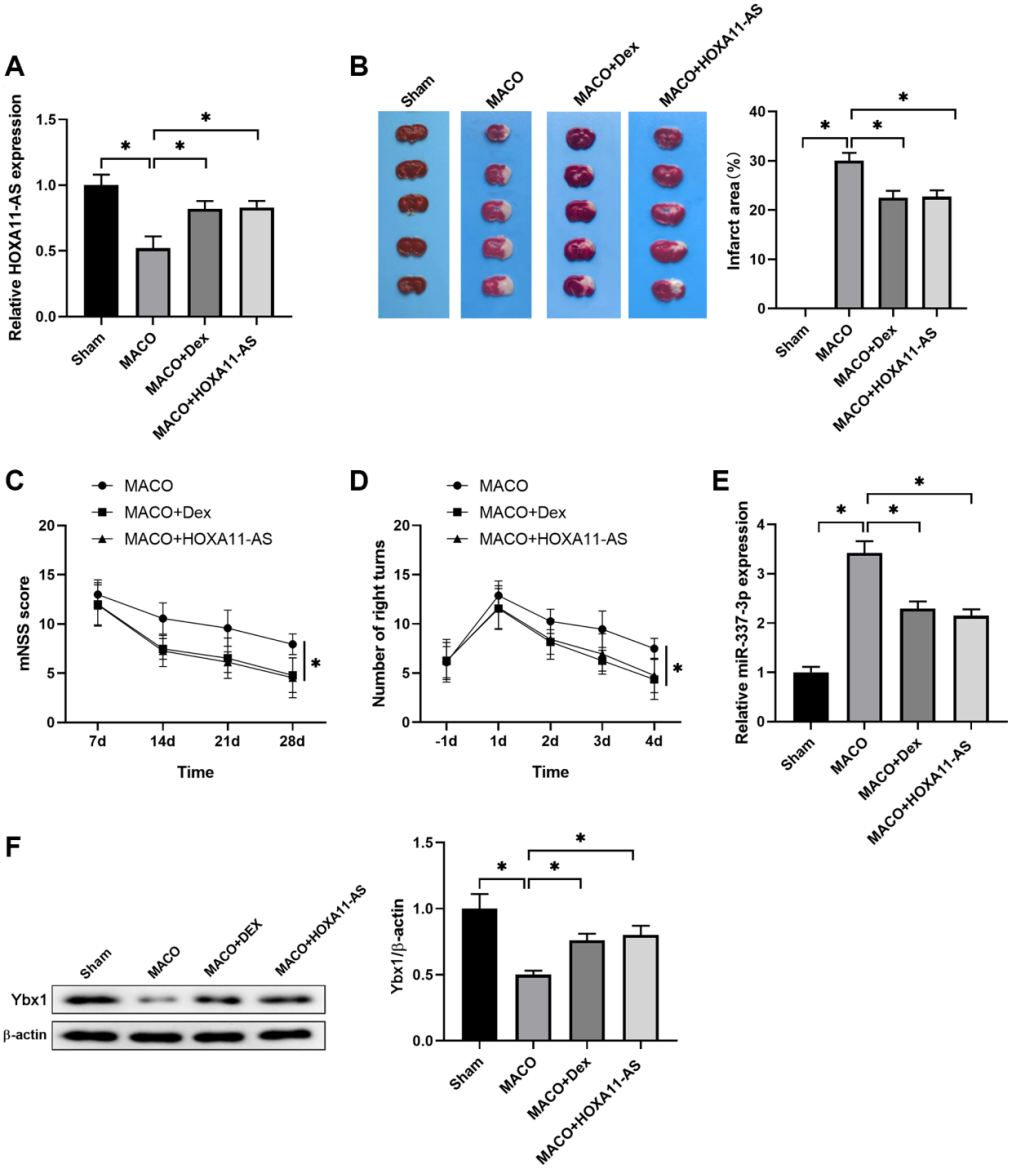
**Dex/HOXA11-AS protected against ischemic damage and improved neurological deficits *in vivo*.** (**A**) The expression of HOXA11-AS was determined by quantitative real-time PCR in mouse brain after MCAO and Dex/HOXA11-AS mimics treatments. *n* = 5 mice for each group. (**B**) Representative illustrations of cerebral sections by TTC staining. Infracted region was stained white. The infarction volume was evaluated as the percentage of infarct area relative to the whole brain. (**C**, **D**) mNSS score (**C**) and numbers of right turns in the Corner test (**D**) in the indicated groups were summarized. *n* = 5 mice for each group. (**E**) The expression of miR-337-3p was determined by quantitative real-time PCR in mouse brain after MCAO and Dex/HOXA11-AS mimics treatments. *n* = 5 mice for each group. (**F**) The levels of Ybx1 in mouse brain after MCAO and Dex/HOXA11-AS mimics treatments were determined by western blot. *n* = 3 for each group. ^*^P < 0.05, compared between the indicated groups.

To verify the mechanisms underlying the neuroprotective effects of Dex, we used Dex and HOXA11-AS mimics to treat mice in the model of ischemia stroke. Dex was intraperitoneally injected to mice and HOXA11-AS mimics was injected with *in vivo* transfection reagent into the lateral ventricle of mice before MCAO operation. The expression of HOXA11-AS was determined by quantitative real-time PCR in mouse brain 24 h after MCAO. Dex and HOXA11-AS mimics treatment increased HOXA11-AS expression, which was down regulated in MCAO mice brain, in comparison to the Sham group (Figure 5A). The brain infarction was detected by TTC staining to visualize the damaged regions for determination of the infarct volume of I/R injury. It showed that Dex and HOXA11-AS mimics treatment significantly decreased the percentages of area with I/R-induced brain infract, compared with the MACO group (Figure 5B). A neurological scoring system and corner test were performed to evaluate neurological deficits after I/R and the beneficial effects of drugs. We found a less neurological score in Dex and HOXA11-AS mimics injected mice, compared with the MACO mice (Figure 5C). It was also noteworthy that Dex and HOXA11-AS mimics injection resulted in better recovery, as evidenced by less right turn in corner test (Figure 5D), which was used to detect unilateral abnormalities of sensory and motor functions in this animal model. Moreover, the increase of miR-337-3p expression induced by MCAO was attenuated by Dex and HOXA11-AS mimics treatments (Figure 5E). Consistently, the decrease of Ybx1 level induced by MCAO was also reversed by Dex and HOXA11-AS mimics treatments (Figure 5F). Collectively, these results indicated that Dex and HOXA11-AS overexpression protected against ischemic damage and improved neurological deficits in MCAO mice model through regulating Ybx1 protein expression.

## DISCUSSION

In this study, we verified that Dex can protect neuronal cells from OGD/R-induced apoptosis. Ischemia and reperfusion injuries are mainly found in acute ischemic stroke, and reperfusion injury is found to be an important cause of secondary brain injury. This study showed that pretreatment of cells with Dex before reperfusion can significantly reduce OGD/R-induced DNA fragmentation, cell viability, and apoptosis.

Cerebral ischemia significantly alters the expression profiles of multiple non-coding RNAs (ncRNAs) species, including microRNAs (miRNAs, 20–25 nt) and long non-coding RNAs (lncRNAs, >200 nt) [34]. These ncRNAs affect the expression of various key elements in cell death and are closely related to neurologic functional disorders [35]. Of all ncRNAs, miRNAs are the most studied as a key mediator of ischemia. Unlike the miRNAs, the function of lncRNAs in stroke pathology is poorly understood. Importantly, more and more experimental evidence has confirmed that lncRNAs act as ceRNAs by competing with the binding of miRNA, and this cross talk has been implicated in various biological processes of disease [17, 18]. In addition, miRNAs participate in various biological processes by regulating their target mRNAs. Therefore, the interaction between lncRNA–miRNA–mRNA may play a key role in the development of ischemia [10, 36]. We found that the HOXA11-AS expression level decreased in OGD/R treated Neuro-2a cells, and Dex increased HOXA11-AS expression in both normal and OGD/R treated cells. Therefore, we aimed to focus on HOXA11-AS, a long non-coding RNA (lncRNA), to study its function and regulatory mechanism in ischemic stroke, as well as its connection with the intervention of Dex. Dexmedetomidine is a highly selective alpha-2 adrenergic receptor agonist and plays its role by binding with receptor on the cell membrane [25, 26]. The possible mechanisms of dexmedetomidine regulating gene expression in cells are not fully elucidated. For example, whether Dexmedetomidine regulates the transcription of HOXA11-AS by influencing transcription factors requires more in-depth studies.

A lot of studies showed that HOXA11-AS overexpression was involved in human tumors progression [14, 16, 37]. There were very few studies about the functional roles of HOXA11-AS in ischemia stroke. We identified that HOXA11-AS expression was abnormally decreased in I/R tissues and cells, which remarkably promoted the cell apoptosis and decreased cell viability. It was important to clarify the underlying molecular mechanism of HOXA11-AS on ischemia stroke and how Dex exerted protective effect through regulating HOXA11-AS. Gain-/loss-of-function studies revealed that HOXA11-AS promoted proliferation, inhibited apoptosis in Neuro-2a cells exposed to OGD/R. Knock down of HOXA11-AS decreased the protective effect of Dex on OGD/R cells, indicating that Dex exerted protective effects on OGD/R exposed Neuro-2a cells at least partially through up-regulation of HOXA11-AS expression.

MiRNA is a class of endogenous non-coding small molecules with a length of 20–24 nt and is widely distributed in the genome of organisms. Mature miRNAs can bind to the 3′-UTR of the target mRNA through base pair complementarity, leading to mRNA degradation and inhibiting the translation process, thereby affecting target gene expression [38]. Changes in miRNAs are associated with neurological diseases [39, 40]. MiRNA regulation has successfully improved a variety of pathologies in animal models [41, 42]. Regarding the treatment of stroke, administration of exogenous miRNAs or silencing of endogenous miRNAs has been shown to affect the severity of cerebral ischemic injury in rodent models [43–45].

We predicted the miRNAs targeted to HOXA11-AS and found changes in expression levels of 8 candidate miRNAs in the cells transfected with pcDNA3.1-HOXA11-AS. miR-337-3p was downregulated when HOXA11-AS was overexpressed. Luciferase assay verified that miR-337-3p expression can be transcriptionally regulated by HOXA11-AS. Treatment with miR-337-3p mimics promoted OGD/R-induced apoptotic cell death and decreased cell viability. HOXA11-AS overexpression markedly decreased the expression of miR-337-3p in cells with OGD/R while HOXA11-AS knockdown increased miR-337-3p expression in OGD/R treated cells. Administration of Dex alone or together with sh-HOXA11-AS both decreased miR-337-3p expression, but Dex alone decreased more significantly. These results showed that HOXA11-AS competed binding to miR-337-3p to inhibit apoptotic cell, which was one of the mechanisms underlying Dex’s neuroprotection. Furthermore, it is worth noting that HOXA11-AS overexpression stimulated the expression of miRNAs including miR-204-5p, miR-320-5p, miR-340-5p, and miR-361-3p. Whether these miRNAs contribute to the protective effects of Dex remains to be further studied.

The Y-box-binding protein-1 (Ybx1) is a member of Y-box protein family binding DNA and RNA, activating a number of genes associated with cell proliferation and cancer development [46]. We found that miR-337-3p might bind to 3′-UTR of Ybx1 mRNA by bioinformatics analysis and it was verified by luciferase reporter assay. miR-337-3p mimics decreased Ybx1 protein expression in both the cells with or without OGD/R, and the different was that OGD/R made the decrease more severely. The treatments with Dex and miR-337-3p inhibitor, separately or simultaneously, increased Ybx1 protein expression in OGD/R induced cells; however, Dex and miR-337-3p inhibitor treatments together increased Ybx1 protein expression much higher. Ybx1 overexpression enhanced cell viability and reduced cell apoptosis induced by OGD/R. These results showed that HOXA11-AS competed with Ybx1 mRNA for binding to miR-337-3p, and Dex protected neuronal cells from apoptosis induced by ischemia/reperfusion through regulating Ybx1 expression.

At last, we verified the mechanism under the neuroprotective effect of Dex using a mice model of ischemia stroke *in vivo*. Dex and HOXA11-AS mimics treatment increased HOXA11-AS expression, improved I/R-induced brain infract volume and improved neurological deficits in MCAO mice model. Dex and HOXA11-AS mimics treatment inhibited the increase of miR-337-3p expression and the decrease of Ybx1 level induced by MCAO. These results indicated that Dex protected against ischemic damage and improved neurological deficits in MCAO mice model through regulating Ybx1 protein expression by increasing the HOXA11-AS level.

Accumulating evidence has suggested that lncRNAs can act as ceRNA by binding to miRNA, thereby affecting the biological processes of cells [15]. Long non-coding RNA H19 regulates FOXM1 expression through competitively binding to endogenous miR-342-3p in gallbladder cancer [47]. HOXA11-AS could act as a ceRNA that repressed miR-146b-5p expression, regulating its downstream target MMP16 in renal cancer [48]. Our study identified for the first time that HOXA11-AS could target miR-337-3p, acting as a ceRNA. Meanwhile, miR-337-3p negatively regulated the expression of its target molecule Ybx1. Moreover, our results suggested that HOXA11-AS promoted cell viability and inhibited cell apoptosis induced by OGD/R, which was linked to increased Ybx1 expression via regulating miR-337-3p. These finding indicated that the HOXA11-AS/miR-337-3p/Ybx1 axis could be a target for OGD/R treatment.

## CONCLUSION

In summary, HOXA11-AS functioned as a ceRNAs and competed with Ybx1 mRNA for directly binding to miR-337-3p, which protected ischemic neuronal death. Dex treatment rescued ischemic damage and improved overall neurological functions *in vivo*. Dex increased cell viability and decreased cell apoptosis induced by ischemia/reperfusion through regulating Ybx1 expression, which was associated with up-regulation in expression of miR-337-3p-targeted lncRNA-HOXA11-AS. Thus, our data suggest a novel mechanism of lncRNA HOXA11-AS as a ceRNA by targeting the miR-337-3p/YBX1 signaling pathway, which is involved in regulating ischemic neuronal death. These findings might provide new insights into developing new strategies for the therapeutic interventions in cerebral ischemic stroke.
